# Closed-Loop Deep Brain Stimulation to Treat Medication-Refractory Freezing of Gait in Parkinson’s Disease

**DOI:** 10.3389/fnhum.2021.633655

**Published:** 2021-03-01

**Authors:** Rene Molina, Chris J. Hass, Stephanie Cernera, Kristen Sowalsky, Abigail C. Schmitt, Jaimie A. Roper, Daniel Martinez-Ramirez, Enrico Opri, Christopher W. Hess, Robert S. Eisinger, Kelly D. Foote, Aysegul Gunduz, Michael S. Okun

**Affiliations:** ^1^Department of Electrical and Computer Engineering, University of Florida, Gainesville, FL, United States; ^2^Norman Fixel Institute for Neurological Diseases and The Program for Movement Disorders and Neurorestoration, University of Florida, Gainesville, FL, United States; ^3^Department of Applied Physiology and Kinesiology, University of Florida, Gainesville, FL, United States; ^4^J. Crayton Pruitt Department of Biomedical Engineering, University of Florida, Gainesville, FL, United States; ^5^Tecnologico de Monterrey, Escuela de Medicina y Ciencias de la Salud, Monterrey, Mexico; ^6^Department of Neurology, University of Florida, Gainesville, FL, United States; ^7^Department of Neuroscience, University of Florida, Gainesville, FL, United States; ^8^Department of Neurosurgery, University of Florida, Gainesville, FL, United States

**Keywords:** freezing of gait (FOG), Parkinson’s disease, pedunculopontine nucleus, closed-loop, deep brain stimulation

## Abstract

**Background**: Treating medication-refractory freezing of gait (FoG) in Parkinson’s disease (PD) remains challenging despite several trials reporting improvements in motor symptoms using subthalamic nucleus or globus pallidus internus (GPi) deep brain stimulation (DBS). Pedunculopontine nucleus (PPN) region DBS has been used for medication-refractory FoG, with mixed findings. FoG, as a paroxysmal phenomenon, provides an ideal framework for the possibility of closed-loop DBS (CL-DBS).

**Methods**: In this clinical trial (NCT02318927), five subjects with medication-refractory FoG underwent bilateral GPi DBS implantation to address levodopa-responsive PD symptoms with open-loop stimulation. Additionally, PPN DBS leads were implanted for CL-DBS to treat FoG. The primary outcome of the study was a 40% improvement in medication-refractory FoG in 60% of subjects at 6 months when “on” PPN CL-DBS. Secondary outcomes included device feasibility to gauge the recruitment potential of this four-lead DBS approach for a potentially larger clinical trial. Safety was judged based on adverse events and explantation rate.

**Findings**: The feasibility of this approach was demonstrated as we recruited five subjects with both “on” and “off” medication freezing. The safety for this population of patients receiving four DBS leads was suboptimal and associated with a high explantation rate of 40%. The primary clinical outcome in three of the five subjects was achieved at 6 months. However, the group analysis of the primary clinical outcome did not reveal any benefit.

**Interpretation**: This study of a human PPN CL-DBS trial in medication-refractory FoG showed feasibility in recruitment, suboptimal safety, and a heterogeneous clinical effect in FoG outcomes.

## Introduction

Medication-refractory, or unresponsive, freezing of gait (FoG) is among the most difficult and disabling symptoms to address in advanced Parkinson’s disease (PD; Moore et al., 2007). The unresponsive FoG phenomenon occurs when PD patients freeze despite optimized dopaminergic medications and improvement in other PD motor symptoms (Espay et al., 2012). Although exercise, physical therapy, and assistive devices have demonstrated clear benefits for FoG (Cosentino et al., 2020), neuromodulation strategies such as deep brain stimulation (DBS) applied in both the globus pallidus internus (GPi) and the subthalamic nucleus (STN) have fallen short in providing therapeutic benefit for medication-refractory FoG and its associated symptoms, such as falling (Deuschl et al., 2006; Okun et al., 2009; Moro et al., 2010b; Williams et al., 2010; Odekerken et al., 2013). Several attempts have been made to alleviate unresponsive freezing by utilizing pedunculopontine nucleus (PPN) and PPN + STN DBS. Overall, these small sample studies have yielded inconclusive findings (Stefani et al., 2007; Strafella et al., 2008; Moreau et al., 2009; Ferraye et al., 2010; Moro et al., 2010a; Acar et al., 2011; Thevathasan et al., 2011, 2018; Wilcox et al., 2011; Khan et al., 2012).

Due to the paroxysmal and heterogeneous nature of FoG, improved clinical outcomes may be achieved with closed-loop DBS (CL-DBS; Rosin et al., 2011; Little et al., 2013, 2016; Rosa et al., 2015, 2017; Piña-Fuentes et al., 2017; Tinkhauser et al., 2017; Arlotti et al., 2018; Molina et al., 2018; Swann et al., 2018; Houston et al., 2019; Velisar et al., 2019; Petrucci et al., 2020). In this technique, stimulation is delivered in response to a specific electrophysiological brain marker that represents periods of activity in which stimulation would be needed (i.e., gait). We aimed to test the safety and feasibility of a closed-loop approach for PPN DBS and to document effects on medication-refractory FoG as well as to collect PPN electrophysiology to serve as our biomarker for CL-DBS. Our strategy also employed conventional open-loop GPi DBS (OL-DBS), which has not been shown to consistently modulate axial symptoms in humans (Ghika et al., 1998; Rocchi et al., 2012; Schrader et al., 2013), to address the levodopa-responsive PD motor symptoms.

## Materials and Methods

### Subjects

**Figure 1:**
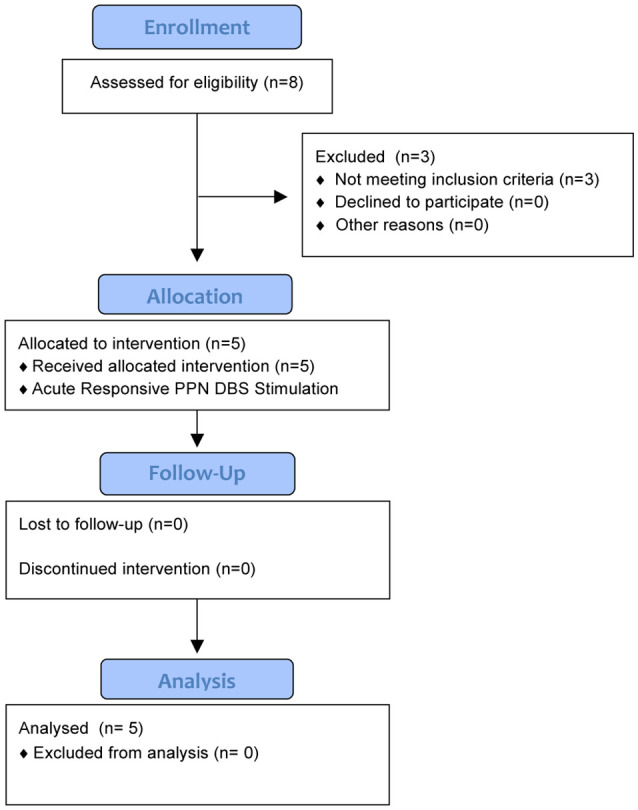
The CONSORT diagram summarizes the study for 6 months of follow-up. Five subjects were enrolled into the study after screening eight potential candidates. Three candidates did not qualify as they did not exhibit five or more freezing of gait (FoG) episodes during the provocation protocol.

This safety and feasibility study was approved for five subjects who all provided written informed consent. The trial was registered with the University of Florida (UF) Institutional Review Board (IRB #201400951) and https://clinicaltrials.gov (NCT02318927), which includes the full inclusion and exclusion criteria. There was also an FDA investigational device exemption (IDE, G140181) in place. An interdisciplinary team at the Norman Fixel Institute for Neurological Diseases at UF screened, reviewed, and approved DBS implantation. Through this process, eight candidates were screened and three failed to meet the inclusion criteria (Figure 1). Subjects were required to have greater than two freezing episodes per month, a score of greater than 1 on item 3 of the Freezing of Gait Questionnaire (FOGQ#3; Giladi et al., 2000), and to exhibit five or more FoG episodes during a provocation screening protocol in the “on” and “off” dopaminergic states. The FoG provocation protocol included stepping in place, walking at a self-selected pace, walking over an obstacle, dual tasking (carrying a tray, answering questions, etc.), turning while walking, and walking through a narrow passage. The off-medication state was defined as a 12-h withdrawal of dopaminergic (L-DOPA) medications, whereas the on-medication state was 45–60 min post-medication administration. The five enrolled subjects had a confirmed medical history of FoG which occurred both “on” and “off” dopaminergic medication, despite aggressive medication optimization by a movement disorders-trained neurologist (Table 1). Furthermore, our subjects had a history of falling, which was confirmed through both extensive chart review and clinical visits.

**Table 1:** Subject demographics.

Subject	Gender	Age (years)	Diagnosis (year of first symptom)	Disease duration at surgery	LEDD (screening/6 months)		UPDRS II (screening /6 months)		UPDRS III off-med/on-GPi DBS only (screening/6 months)		UPDRS III on-med/on-GPi DBS only (screening/ 6 months)		UPDRS IV (screening/ 6 months)	
1	F	50	2009 (2001)	6 (14)	502.5	577.5	26	22	38	24	23	19	5	7
2	M	52	2008 (2006)	7 (9)	622.5	607.5	23	17	31	37	21	31	5	3
3	M	74	2008 (2005)	7 (10)	275	350	30	40	42	57	34	52	5	6
4	M	60	1995 (1994)	20 (21)	625	625	24	21	49	46	30	41	14	5
5	F	60	2013 (2011)	2 (4)	427.5	405	35	33	40	48	28	42	5	6

*UPDRS scores were performed On-GPi DBS only. PPN DBS was not activated during these scales. *UPDRS*, Unified Parkinson’s Disease Rating Scale; *LEDD*, levodopa equivalent daily dose; *GPi*, globus pallidus internus; *DBS*, deep brain stimulation*.

### Assessments and Device Programming

Information regarding device and surgical implantation can be found in Molina et al. (2020). Briefly, electrodes were implanted bilaterally in both the GPi (Medtronic 3387 leads) and PPN (Medtronic 3389 leads) and the implantation procedure was divided into three stages. In the first stage, two leads (PPN + GPi) were unilaterally implanted; in the second stage of the operation 2–4 weeks later, the other two leads were implanted in the contralateral hemisphere. In one subject (subject 3), two PPN leads were placed in stage 1 and the two GPi leads in stage 2. In the final stage, which occurred approximately 4 weeks after stage 2, GPi DBS leads were connected to one Medtronic Activa PC + S (Medtronic PLC, Minneapolis, MN, USA), the implantable neurostimulator (INS), and secured in a sub-clavicular pocket, while the PPN leads were connected to a separate Activa PC + S. Postoperative CT images co-registered with preoperative MRI were used to confirm the postoperative position of the active contacts (Supplementary Table 1). At the end of the study, patients whose systems were not explanted kept both implantable neurostimulators. If the patient and clinician decided not to use the PPN leads after study conclusion, they were deactivated.

Monthly visits were initiated 4 weeks after the last surgical phase and occurred until month 10, followed by visits at months 12 and 18. During monthly visits, the subjects performed clinical evaluations and biomechanical studies while “off” and then “on” L-DOPA medications. Every month included the FOGQ, the Gait and Falls Questionnaire (GFQ; Giladi et al., 2000), the Activities-Specific Balance Confidence Scale (ABC; Powell and Myers, 1995), the Parkinson’s Disease Quality of Life Questionnaire (PDQ)—39 (Peto et al., 1998), and the Unified Parkinson’s Disease Rating Scale (UPDRS; Fahn and Elton, 1987).

The primary outcome variable was a comparison of the preoperative number of FoG episodes vs. the number of FoG episodes at 6 months post-DBS at the optimized GPi OL-DBS and PPN CL-DBS settings (Table 2). Two tasks were used to quantify the primary outcome of the study: (1) stepping in place (SIP; Nantel et al., 2011); and (2) gait at a self-selected pace (SSP). SIP was collected first during visits. The SIP protocol consisted of three trials of 90 s of SIP in which the subjects were asked to raise their legs alternately at a self-selected pace. During SSP, the subjects were asked to walk at their comfortable, preferred pace over-ground across an 8-m walkway a total of 10 times. Changes in stamina and disease state necessitated a normalized FoG count. The “on” and “off” medication condition FoG counts were normalized to the number of trials from each task, which ranged from 1 to 6 (mean, 3.4) for SIP and from 10 to 22 (mean, 9.6) for SSP, in each respective medication state, and were then summed. The percent improvement was then calculated from the total combined count. Not all subjects completed the tasks at each month in each condition due to the inability to perform the tasks off medication (subject 3) or due to fatigue. In order to meet the predetermined primary outcome variable, 60% of the subjects (three of five) were required to show a greater than 40% improvement from baseline on the combined “on” and “off” medication normalized FoG counts. An independent, blinded movement disorders-trained neurologist reviewed video recordings of the subjects performing the FoG provocation protocol and labeled freezing events (Nutt et al., 2011).

**Table 2:** Stimulation parameters at 6 months.

Nucleus	GPi				PPN					
Subject	Active contacts	Amp. (V)	PW (μs)	Freq. (Hz)	PPN recording side	Recording contact	Active contacts	Amp. (V)	PW (μs)	Freq. (Hz)
1	2-C+	3.4	90	185	Right	8–10	1-C+	0.8	60	65
	10-C+	2.2	90	185			9-C+	0.8	60	65
2	1-C+	2.1	90	180	Left	0–2	1-C+	0.7	60	65
	10-C+	2.2	90	180			9-C+	0.7	60	65
3	2-C+	3.5	90	135	Right	8–10	1-C+	0.8	60	65
	9–10-C+	2.1	90	135			9-C+	0.8	60	65
4	2-C+	1.8	100	135	Left	0–2	1-C+	0.4	60	65
	11-C+	2.8	100	135			9-C+	0.4	60	65
5	1–2+	2.6	90	180	Left	1–3	2-C+	0.6	60	65
	9–10+	2.6	60	180			9-C+	0.6	60	65

*Amp., Amplitude; PW, Pulse width; Freq., Frequency. PPN recording side is the electrode side which contains the recording contacts for the ambulation biomarker. Recording contacts are the sensing contacts on the respective recording electrode. Active contacts are those that delivered stimulation*.

Secondary outcome measures included feasibility of recruitment, safety, and adverse events. All adverse events (AEs) were recorded and scored by a physician to determine whether they were related to the study procedure. AEs were scored for severity and outcome. Other outcome variables were the changes from baseline to 6 and 12 months on the FOGQ, GFQ, ABC, PDQ, Berg Balance Scale (BBS; Berg et al., 1992), UPDRS III, total UPDRS scores, and L-DOPA response, which we defined as the difference between the UPDRS-III off and on medication total score divided by the UPDRS-III off medication total score.

During the monthly visits, electrophysiology data from bilateral GPi and PPN were collected using the Activa PC + S. The neural data were aligned to external sensors (Trigno, Delsys Inc., Natick, MA, USA) and video recordings, and subsequently used to develop the PPN CL-DBS paradigm. Gait performance was assessed using 3D motion capture (Vicon Motion Systems, Oxford, UK) and spatiotemporal parameters of interest were calculated using custom MATLAB software (2016a Mathworks, Natick, MA, USA) based on definitions from Whittle’s Gait Analysis (Levine et al., 2012). Participants wore retroreflective markers on the lower extremity to measure gait speed and stride length, which were calculated based on standard definitions. Specifically, gait speed was the average stride velocity across an 8-m walkway when participants were walking at a “steady” pace (i.e., not accelerating or decelerating), and stride length was the horizontal distance between subsequent heel strikes along the line of progression.

### Closed-Loop Implementation

From month 4 onward, we used the Medtronic Nexus-D platform (Afshar et al., 2013), which is a telemetry wand that allows a direct interface to the DBS INS and enables real-time neural data streaming to a host computer. This platform facilitated not only the acquisition of the neural data needed to identify a CL-DBS biomarker but also the delivery of acute PPN CL-DBS in the laboratory setting. CL-DBS stimulation was delivered to the PPN and was triggered by an increase in power of the 1- to 8-Hz band from the PPN region (Molina et al., 2020), which was identified to modulate most consistently with gait.

The acute PPN CL-DBS paradigm was used to establish the parameters for long-term PPN CL-DBS, in which the subjects received PPN CL-DBS outside of the laboratory. Long-term PPN CL-DBS was delivered *via* the Nexus-E firmware, which allowed a similar Nexus-D operation, but was completely embedded within the Activa PC + S (i.e., the INS). However, the Activa PC + S onboard classifier uses a linear discriminant analysis approach, which permits the use of only two power bands with a minimum and a maximum bandwidth of 5 and 32 Hz, respectively. Therefore, the center frequency of our CL-DBS power band was 5 Hz with a bandwidth of 5 Hz (i.e., 2.5–7.5 Hz) to capture our 1- to 8-Hz gait signal within the PPN. Once the 2.5- to 7.5-Hz signal exceeded a predefined threshold, which was derived from the training data during off-stimulation periods and was extracted from a receiver operating characteristic curve (ROC) that maximized specificity and sensitivity, PPN stimulation would initiate and consistently stimulate for 3.5 s after the onset of detection. By sweeping through various hold times, 3.5 s was chosen since it maximized the ROC area under the curve (AUC), which was delineating walking and rest. Longer hold times did not increase the AUC (i.e., the performance of the detector). Since the PPN gait signal did not always produce a large or sustained power increase and was overcome with noise from the stimulation pulse, we defined hold times from a histogram of inter-detection intervals during walking (Supplementary Figure 1).

For both acute and long-term PPN CL-DBS, a 65-Hz frequency setting with a 60-μs pulse width was chosen for all subjects based on previous FoG studies (Mazzone et al., 2005; Plaha and Gill, 2005; Stefani et al., 2007; Ferraye et al., 2010; Moro et al., 2010a; Thevathasan et al., 2011) and also empirical programming that yielded minimal stimulation artifacts. Both Nexus-E and Nexus-D solutions sensed unilaterally and delivered stimulation bilaterally from 0 V to the individual target’s therapeutic voltage (Table 2, PPN settings). The side chosen for unilateral PPN sensing was based on which nuclei had the more robust gait biomarker. For GPi stimulation, patients underwent standard-of-care DBS parameter optimization. The same clinical settings were used for GPi stimulation throughout either acute or long-term PPN CL-DBS. For subjects 1 and 5, long-term CL-PPN DBS was initiated at months 12 and 8, respectively. Therefore, secondary outcome measures at month 18 for subject 1 and at months 9, 10, and 12 for subject 5 were all conducted when the patient was on CL-PPN DBS (Supplementary Table 2).

### Statistical Analysis

Significant changes in the primary outcome variable (i.e., FoG count), stride length, velocity, and BBS between baseline and month 6 were evaluated using a repeated measures ANOVA. To evaluate changes between screening, 6 months, and 12 months, a mixed model was used for the following outcome variables: FOGQ, FOGQ#3, GFQ, PDQ-39 total score, PDQ-39 mobility subscore, ABC, total UPDRS (both on and off medication), UPDRS III (both on and off medication), levodopa (L-DOPA) response, and medication doses (i.e., levodopa equivalent daily dose, LEDD). We chose a mixed model instead of a repeated measures ANOVA since subject 2 is missing data from month 12 due to device explantation. *Post hoc* pairwise comparisons were adjusted with Bonferroni correction. Significance was defined as a *p*-value < 0.05. All statistics were completed in R 3.5.2. Additionally, given the small sample size and variable follow-ups, we have focused on individual outcomes as well as group outcomes at screening, 6 months, and 12 months.

## Results

### Feasibility and Safety

**Figure 2:**
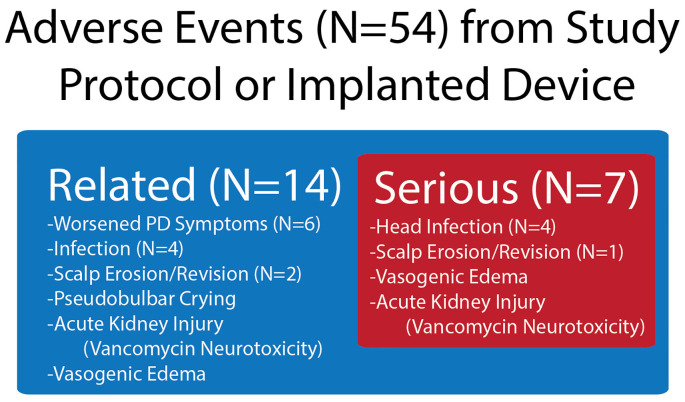
Device- and procedure-related adverse events (AEs) in the study, which were drawn from all AEs. Shown in the figure are the number of AEs related to the device or study protocol and from the related number of those AEs that were severe.

The feasibility of recruiting patients with both “on” and “off” medication FoG was achieved. However, the safety profile was suboptimal, with a 40% device explantation rate due to infection. Of 54 AEs reported, 14 were related to either the implanted device or to the study procedure (Figure 2). From the related AEs, seven were determined to be severe. The numbers of infection and scalp erosion events reflect the initial event and subsequent difficulty with wound healing, which occurred in two subjects, 2 and 4. Subjects 2 and 4 were withdrawn from the study before long-term closed-loop PPN stimulation could be implemented and had their entire DBS systems explanted at months 12 and 16, respectively, due to infections. Thus, the final follow-up visits for subjects 2 and 4 were months 10 and 12, respectively. Subjects 3 and 5 reported worsening of symptoms, specifically gait and balance impairments, immediately following the first lead implantation. Subject 3’s worsening subsided before undergoing his second bilateral implantation; however, he was lost to follow-up after month 12. Vasogenic edema was observed by imaging following the first surgical phase (PPN and GPi left lead implantation) in subject 5, which may have led to the worsening of PD symptoms pertaining to gait and balance that persisted throughout the study. Subject 1 experienced a worsening of gait and balance following the second surgical phase (PPN and GPi right lead implantation), which persisted throughout the study.

### Primary Outcome Variable—FoG Episode Counts

The primary outcome variable was met in three of the five subjects who exhibited a greater than 40% improvement in the number of FoG episodes from baseline to 6 months when on acute PPN CL-DBS (Table 3). There was no significant difference between the pre-DBS and month 6 FoG counts at the group level (*F*_(1,4)_ = 0.053, *p* = 0.0829).

**Table 3:** Primary outcome of the FoG episode count.

	FoG count			No. of SIP trials (pre-DBS/6 months				No. of SSP trials (pre-DBS/6 months			
Subject	Pre-DBS	Month 6	Improvement (%)	Off		On		Off		On	
1	4.3	7.0	−63	3	3	2	3	10	10	6	10
2	1.8	0.2	89	5	0	4	3	10	10	0	0
3	6.7	2.0	70	0	0	3	1	0	0	10	10
4	4.1	0.3	93	3	0	3	6	10	10	10	12
5	11.2	16.4	−46	3	6	1	6	10	10	5	10

*The FoG count, which was our primary outcome variable, was completed by a movement disorder neurologist who was blinded to all stimulation conditions. Subjects 2, 3, and 4 met the 40% improvement criteria for a positive trial (highlighted in the table). Counts were normalized to the number of trials and combined in the “on” and “off” L-DOPA state*.

### Secondary Outcome Measures

#### Group Analysis

**Figure 3:**
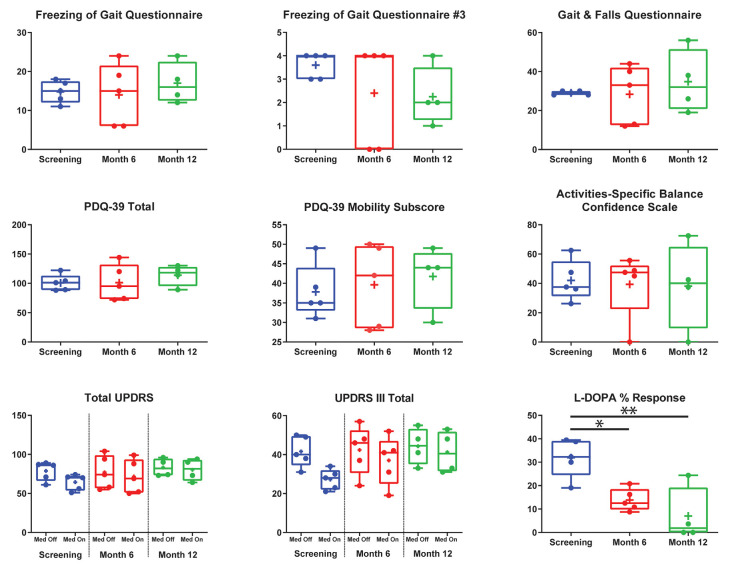
Group summary of clinical outcome measures. This figure addresses the entire cohort before deep brain stimulation (DBS) implantation (*blue*) after 6 (*red*) and 12 (*green*) months post-implantation on the Freezing of Gait Questionnaire (FOGQ), FOGQ#3, Gait and Falls Questionnaire (GFQ), Parkinson’s disease (PD) Quality of Life Questionnaire—3 (PDQ-39) total, PDQ-39 mobility, Activities-Specific Balance Confidence Scale (ABC), total Unified Parkinson’s Disease Rating Scale (UPDRS), UPRS-III, and L-DOPA percent response. All graphs are standard box plots, with *dots* indicating individual scores, and *plus signs* indicating means. FOGQ question #3 was “Do you feel that your feet get glued to the floor while walking, making a turn or when trying to initiate walking (freezing)?” *Off Med*, off dopaminergic medication; *On Med*, on dopaminergic medication. All clinical measures were performed on globus pallidus internus (GPi) open-loop DBS. Pedunculopontine nucleus DBS was not activated during secondary outcome measures, except at month 12 for subject 5. *p < 0.05, **p < 0.01.

**Figure 4:**
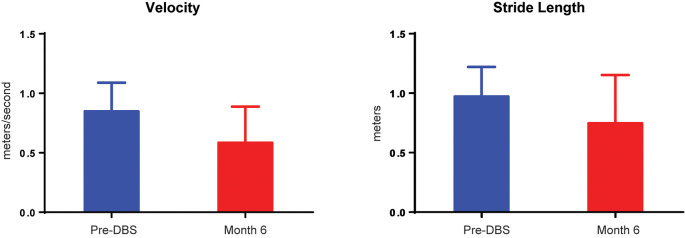
Velocity and stride decrements in five subjects. A comparison of pre-DBS (*blue*) and 6 months (*red*) demonstrated no significant changes in gait velocity (in meters per second) or stride length (in meters). All data are plotted as μ + SD.

There were no significant differences for any measure between pre-DBS, month 6, and month 12 (Figure 3), except a worsening of L-DOPA response (*F*_(2,7.32)_ = 12.83, *p* < 0.01), in which *post hoc* comparisons demonstrated a significant decrease from pre-DBS to month 6 (*t* = 3.77, *p_adj._* = 0.020) and pre-DBS to month 12 (*t* = 4.74, *p_adj._* = 0.005). Additionally, there was no significant difference found for LEDD between any time point (*F*_(2,7.02)_ = 0.38, *p* = 0.70). Gait metrics were compared between baseline and 6 months while the subjects were on levodopa (Figure 4). Overall, the subjects’ velocities (baseline, 0.84 ± 0.24; month 6, 0.59 ± 0.30; *F*_(1,4)_ = 4.07, *p* = 0.11) and stride lengths (baseline, 0.97 ± 0.24; month 6, 0.75 ± 0.41; *F*_(1,4)_ = 3.29, *p* = 0.14) did not change from baseline to 6 months.

#### Individual Outcomes

**Figure 5:**
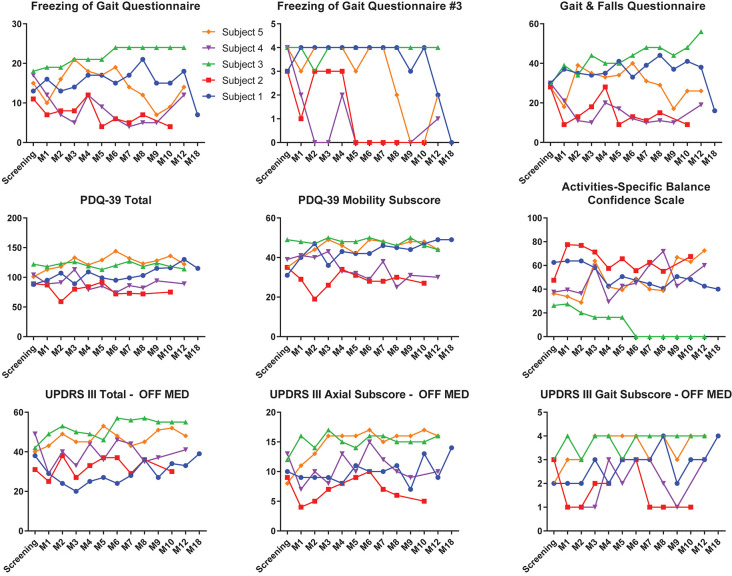
Clinical outcome scores of each individual subject pre-deep brain stimulation (DBS) until the last available follow-up. Individual outcomes for each individual subject on the Freezing of Gait Questionnaire (FOGQ), FOGQ#3, Gait and Falls Questionnaire (GFQ), PD Quality of Life Questionnaire—3 (PDQ-39) total, PDQ-39 mobility, Activities-Specific Balance Confidence Scale (ABC), Unified PD Rating Scale III (UPDRS-III) off dopaminergic medication, UPDRS-III axial subscore (items 18, 27–30) off medication, and UPDRS-III gait subscore (item 29) off medication. Subjects 1 and 5 were on long-term pedunculopontine nucleus (PPN) closed-loop DBS (CL-DBS) during their 18- and 9- to 12-month visits, respectively. All other subjects did not have their PPN DBS activated. GPi open-loop DBS (OL-DBS) was activated during all outcomes.

Individual clinical measures prior to DBS and throughout the entirety of the study are summarized in Figure 5. At 6 months, subjects 2 and 4 experienced improvements from screening to their last visit and month 9, respectively, in the FOGQ, FOGQ#3, GFQ, PDQ-39 total score, PDQ-39 mobility subscore, and ABC (Figure 5). Subject 1, who initiated long-term PPN CL-DBS after her month 12 visit, improved in FOGQ, FOGQ#3, and GFQ, from both baseline and month 12 at month 18, or after 6 months of long-term PPN CL-DBS. Furthermore, she slightly improved in her PDQ-39 total score and PDQ-39 mobility subscore from month 12 to 18. All other subscores worsened or remained the same from both baseline and month 12 at month 18 (Figure 5). Subject 5 began long-term PPN CL-DBS after month 8, in which she improved from month 8 to 9 in FOGQ, FOGQ#3, GFQ, ABC, and the UPDRS-III gait subscore; however, these initial improvements were not consistent across these and all other subscores up until her last visit (Figure 5).

## Discussion

We present the feasibility, safety, and clinical results of a PPN CL-DBS GPi OL-DBS trial in five individuals with unresponsive freezing of gait. Recruitment was feasible and the primary outcome was met; however, it resulted in a suboptimal safety profile, which included a 40% explantation rate due to delayed infection. Since other DBS studies have successfully applied four-lead approaches (PPN + STN; Stefani et al., 2007; Mazzone et al., 2009; Ferraye et al., 2010), it is likely that our specific atypical and fragile patient population of markedly disabled unresponsive freezers in the early to moderate stages of their disease were negatively impacted by this surgical approach. Another study that implanted bilateral PPN leads in patients with FoG experienced significant surgical side effects in two of six patients, leading to one explantation (Welter et al., 2015). This evidence, combined with our previous experiences (Okun et al., 2009), indicates that the choice of two sets of bilateral leads may be high risk in patient populations with atypical PD symptoms (refractory freezes) who are at a greater risk of falling. The primary clinical outcome of greater than 40% improvement in medication-refractory FoG in three of five subjects was achieved at 6 months when “on” acute PPN CL-DBS. However, the group analysis of the change in FoG counts from pre-DBS to month 6 on acute PPN CL-DBS did not reveal a significant benefit.

An important aspect to the study was the rigid inclusion criteria. During the planning phase, we reasoned that if available medications or DBS could greatly improve or resolve “off” medication FoG, then PPN therapy would not be necessary. Therefore, the more critical need for the PD community was a therapy targeting medication-refractory FoG, which usually presents with patients displaying both on-medication and off-medication FoG. One potential issue with this selection criterion is that PD patients with on-medication FoG may be more clinically fragile and suffer from more comorbidities. Thus, it is likely that the culmination of the atypical patient population (i.e., medication-refractory FoG patients and high-risk fallers) and the four-lead approach, which had immediate impacts in three of the five patients in the study and persistent effects in two of five likely contributed to our suboptimal safety profile.

There were no significant differences between baseline and 6 or 12 months in any secondary outcome variables, except a decline in L-DOPA response. Although this may point to disease progression, we believe that this is due to the assessments being performed under GPi stimulation or a lesion effect from surgery. Additionally, the gait metrics were not significantly different from baseline and were within the range of metrics from another large cohort study (*N* = 310; Hass et al., 2012), in which the subjects of this study were within the PDQ mobility subscore, UPDRS motor subscore, and disease duration range of the larger cohort.

All outcome variables, besides the FoG counts, were performed when patients were on only GPi OL-DBS settings, with the exception of month 18 for subject 1 and months 9, 10, and 12 for subject 5; thus, these effects were primarily driven by the GPi OL-DBS settings. For subject 1, PPN CL-DBS did improve the subjective measures of failing and freezing, including the FOGQ, GFQ, and PDQ-39. However, on the objective measurements of UPDRS-III, PPN CL-DBS led to a worsening of the total score as well as the axial (items 18, 27–30) and gait (item 29) subscores from 12 to 18 months (Figure 5). Yet, this worsening may have stemmed from disease progression rather than PPN CL-DBS over those 6 months or from the paroxysmal nature of FoG. Subject 5 experienced an alleviation of scores on the FOGQ and GFQ after 1 month of PPN CL-DBS; however, the effects were inconsistent at her remaining visits (Figure 5). Overall, the effects of PPN CL-DBS have been proven to be modest in these two subjects.

This article established a PPN CL-DBS paradigm driven by a gait biomarker, which was defined as an increase in 1- to 8-Hz power within the PPN (Molina et al., 2020). An increase in low-frequency oscillations (7–10 Hz) within the PPN has been previously described in patients with PD during gait (Thevathasan et al., 2012). A potential limitation of this biomarker is that it may be due to movement artifact rather than gait; however, we do not believe that this is the case. During gait, we did not observe a broadband increase in PPN or GPi activity (Supplementary Figure 2). Furthermore, if the signal we were identifying was in fact an artifact produced from the device, it would also be observed within the GPi recordings.

Various continuous stimulation PPN DBS studies have produced varied results, and there has been recent cautious optimism about the possibility of addressing FoG (Stefani et al., 2007; Strafella et al., 2008; Moreau et al., 2009; Moro et al., 2010a; Acar et al., 2011; Thevathasan et al., 2011; Wilcox et al., 2011; Khan et al., 2012). Although there are published PPN studies with acute improvement, most subjects have failed to maintain positive long-term outcomes (Mestre et al., 2016), similar to the two subjects in this study who underwent long-term PPN CL-DBS. Within other studies that selected patients with unresponsive FoG (Thevathasan et al., 2011) or patients with gait disturbances in progressive supranuclear palsy (Doshi et al., 2015), benefits in gait and balance until 24 and 18 months, respectively, were perceived. However, their cohorts only received bilateral PPN stimulation, whereas in our subjects, we may have perceived inconsistent benefits due to co-stimulation of the GPi (Thevathasan et al., 2018). Our lack of chronic benefit and heterogeneous clinical results in our two long-term PPN subjects was similar to other studies delivering stimulation to multiple targets (Ferraye et al., 2010; Goetz et al., 2019). Furthermore, the failure to maintain benefit from PPN DBS could be a result of many factors including patient selection, microlesion effects, balance dysfunction, disease progression, and electrode locations (Goetz et al., 2019) as well as whether continuous vs. closed-loop DBS programming approaches have been applied.

There were several limitations with the approach in this study. First, eliciting FoG in the laboratory setting is difficult (Nieuwboer et al., 2001; Giladi and Nieuwboer, 2008). Additionally, many FoG episodes are ambiguous and can lead to labeling difficulty, even for experienced movement disorders-trained neurologists. Second, we developed and implemented a new CL-DBS algorithm without knowing whether there would be a consistent and robust physiological signal, which would ultimately define who would undergo long-term PPN CL-DBS. Accomplishing this task in a human population as well as with a new DBS device (Activa PC + S) was non-trivial, and we explored many possible algorithms to identify the best approach for each patient based on their individual physiology. The study sample was small and lacked a control group, which would be helpful to judge the clinical results. Furthermore, we did not test the primary outcome variable with GPi-DBS turned both on and off in order to elucidate the effects of PPN-DBS. Finally, though all five patients met the criteria for a diagnosis of PD, it is possible given the disease progression and complications that some of this cohort may have had other parkinsonism-related diagnoses. Without postmortem confirmation from any of the patients, we cannot be certain of the diagnoses.

In conclusion, FoG as a paroxysmal phenomenon provides an ideal framework for closed-loop DBS; however, the approach resulted in heterogeneous clinical and physiological outcomes and did not reach a reasonable safety standard to warrant a follow-up study. A safer approach may be to limit patient selection to “off” freezers only while implanting and developing closed-loop DBS in a single deep brain target (e.g., PPN).

## Data Availability Statement

Data will be made available upon reasonable request.

## Ethics Statement

This study involved human participants and was reviewed and approved by the University of Florida Institutional Review Board. The patients/participants provided their written informed consent to participate in this study.

## Author Contributions

RM contributed to the conceptualization, methodology, software, formal analysis, investigation, resources, data curation, writing of the original draft, review and editing, and visualization. CH helped with the conceptualization, methodology, review and editing, supervision, and funding acquisition. SC performed formal analysis, revisions, and review and editing. KS, AS, and JR helped with the validation, formal analysis, investigation, and review and editing. DM-R performed formal analysis, data curation, and review and editing. EO helped with the methodology, software, formal analysis, and investigation. CWH and RE helped with the formal analysis and review and editing. KF, AG, and MO contributed to the conceptualization, methodology, resources, supervision, and funding acquisition. All authors contributed to the article and approved the submitted version.

## Conflict of Interest

CH receives research funding from NIH and the Michael J. Fox Foundation. AS receives research funding from the American Society of Biomechanics and the Elaine C. Pidgeon Neurology Research Fund. JR receives research funding from the Department of Defense. KF receives research and fellowship support from Medtronic, St. Jude, Boston Scientific, NeuroPace, and Functional Neuromodulation. AG receives device donations from Medtronic, served on an advisory board for the Michael J. Fox Foundation, and receives research grants from the NIH, NSF, and DARPA. MO serves as a consultant for the National Parkinson Foundation, and has received research grants from the NIH, NPF, the Michael J. Fox Foundation, the Parkinson Alliance, Smallwood Foundation, the Bachmann-Strauss Foundation, the Tourette Syndrome Association, and the UF Foundation. He has previously received honoraria, but in the past >60 months has received no support from industry. He has received royalties for publications with Demos, Manson, Amazon, Smashwords, Books4Patients, and Cambridge (movement disorders books); is an associate editor for New England Journal of Medicine Journal Watch Neurology; and has participated in CME and educational activities on movement disorders sponsored by PeerView, Prime, QuantiaMD, WebMD, MedNet, Henry Stewart, and by Vanderbilt University. The institution and not MO receives grants from Medtronic, Abbvie, Allergan, and ANS/St. Jude, and the PI has no financial interest in these grants. The remaining authors declare that the research was conducted in the absence of any commercial or financial relationships that could be construed as a potential conflict of interest.

## Supplementary Material

SUPPLEMENTARY FIGURE 1 Example of local field potential recordings from PPN. **(A)** Real-time detection of gait demonstrating the intermittent spiking nature of the gait feature. Inter-detection intervals defined the 3.5 s hold out. Blue areas denote when the subject was walking. **(B)** Fully implemented responsive PPN-DBS. The top panel is the right and left foot acceleration, the middle panel is the raw PPN signal, and finally the bottom panel is the gait feature band. The algorithm successfully detected walking, and subsequently turned on stimulation, maintained stimulation for the majority of the walking task, and turned stimulation back off when ambulation stopped.

SUPPLEMENTARY FIGURE 2 Spectrograms from GPi and PPN in one participant. **(A)** One representative spectrogram of GPi activity before and after the onset of walking (*n* = 20 trials). The onset of walking is denoted by the black vertical line at 0 s. **(B)** Spectrogram of PPN activity before and after the onset of walking. Note the increased power within the PPN is confined to lower frequencies rather than a broadband sharp increased across frequencies. Similar, no artifact is present within the GPi recordings from the same trials.

SUPPLEMENTARY TABLE 1 Lead locations of the active contacts.

SUPPLEMENTARY TABLE 2 Stimulation protocol at each month.
