# Supplementary material for: Closed-Loop Deep Brain Stimulation to Treat Medication-Refractory Freezing of Gait in Parkinson’s Disease
Source: Front Hum Neurosci. 2021 Mar 1;15:633655. doi: 10.3389/fnhum.2021.633655 (PMC7959768; doi:10.3389/fnhum.2021.633655)
Supplement: SUPPLEMENTARY FIGURE 1 — Example of local field potential recordings from PPN. (A) Real-time detection of gait demonstrating the intermittent spiking nature of the gait feature. Inter-detection intervals defined the 3.5 s hold out. Blue areas denote when the subject was walking. (B) Fully implemented responsive PPN-DBS. The top panel is the right and left foot acceleration, the middle panel is the raw PPN signal, and finally the bottom panel is the gait feature band. The algorithm successfully detected walking, and subsequently turned on stimulation, maintained stimulation for the majority of the walking task, and turned stimulation back off when ambulation stopped. [file Table_1.DOCX]

Supplementary Table 1. Lead locations of the active contacts.

| Nucleus | Left GPi  Right GPi | | |  | Left PPN  Right PPN | | | |
| --- | --- | --- | --- | --- | --- | --- | --- | --- |
| Subject | Active Contacts | x  (mm) | y  (mm) | z  (mm) | Active Contacts | x  (mm) | y  (mm) | z  (mm) |
| 1 | 2-C+  10-C+ | -22.4  18.5 | 2.8  5.3 | -0.1  1.0 | 1-C+  9-C+ | -7.4  3.9 | -19.1  -18.6 | -11.0  -12.1 |
| 2 | 1-C+  10-C+ | -22.6  21.9 | 2.2  4.0 | -3.1  0.2 | 1-C+  9-C+ | -6.7  6.6 | -18.1  -19.5 | -14.5  -18.0 |
| 3 | 2-C+  9-10-C+ | -26.4  20.4  20.9 | 2.2  3.1  3.4 | 2.1  -3.1  -0.1 | 1-C+  9-C+ | -7.6  6.0 | -20.9  -19.3 | -11.5  -11.0 |
| 4 | 2-C+  11-C+ | -23.4  22.8 | 6.0  3.4 | 0.7  -3.0 | 1-C+  9-C+ | -10.8  4.6 | -17.3  -18.0 | -12.6  -14.0 |
| 5 | 1-2+  9-10+ | -20.8  -21.3  20.6  20.8 | 1.7  2.8  1.4  3.1 | -0.1  2.7  -0.5  2.1 | 2-C+  9-C+ | -7.0  6.0 | -18.9  -20.1 | -10.9  -11.2 |

X, y, and z are coordinates in AC-PC space of the active contact. If bipolar stimulation, the location of both contacts are provided.
