# Supplementary material for: Closed-Loop Deep Brain Stimulation to Treat Medication-Refractory Freezing of Gait in Parkinson’s Disease
Source: Front Hum Neurosci. 2021 Mar 1;15:633655. doi: 10.3389/fnhum.2021.633655 (PMC7959768; doi:10.3389/fnhum.2021.633655)
Supplement: SUPPLEMENTARY FIGURE 2 — Spectrograms from GPi and PPN in one participant. (A) One representative spectrogram of GPi activity before and after the onset of walking (n = 20 trials). The onset of walking is denoted by the black vertical line at 0 s. (B) Spectrogram of PPN activity before and after the onset of walking. Note the increased power within the PPN is confined to lower frequencies rather than a broadband sharp increased across frequencies. Similar, no artifact is present within the GPi recordings from the same trials. [file Table_2.DOCX]

| **Supplementary Table 2.** Stimulation Protocol at Each Month | | | | | | | | | | | | |
| --- | --- | --- | --- | --- | --- | --- | --- | --- | --- | --- | --- | --- |
| Subject | M1 | M2 | M3 | M4 | M5 | M6 | M7 | M8 | M9 | M10 | M12 | M18 |
| 1 |  |  |  |  |  |  |  |  |  |  |  |  |
| 2 |  |  |  |  |  |  |  |  |  |  |  |  |
| 3 |  |  |  |  |  |  |  |  |  |  |  |  |
| 4 |  |  |  |  |  |  |  |  |  |  |  |  |
| 5 |  |  |  |  |  |  |  |  |  |  |  |  |
| **Pink:** GPi OL-DBS only (individual gait feature extraction)  **Blue:** GPi OL-DBS and acute CL-PPN DBS in the lab setting *only* (i.e. during primary outcome variable)  **Green:** GPi OL-DBS and chronic CL-PPN DBS (i.e. during both primary and secondary outcome measures)  **Black:** Patient was not seen at this month | | | | | | | | | | | | |
