# Supplementary figures and images for: Closed-Loop Deep Brain Stimulation to Treat Medication-Refractory Freezing of Gait in Parkinson’s Disease
Source: Front Hum Neurosci. 2021 Mar 1;15:633655. doi: 10.3389/fnhum.2021.633655 (PMC7959768; doi:10.3389/fnhum.2021.633655)

**A**

Walking    Detection    Band Power    PPN Threshold

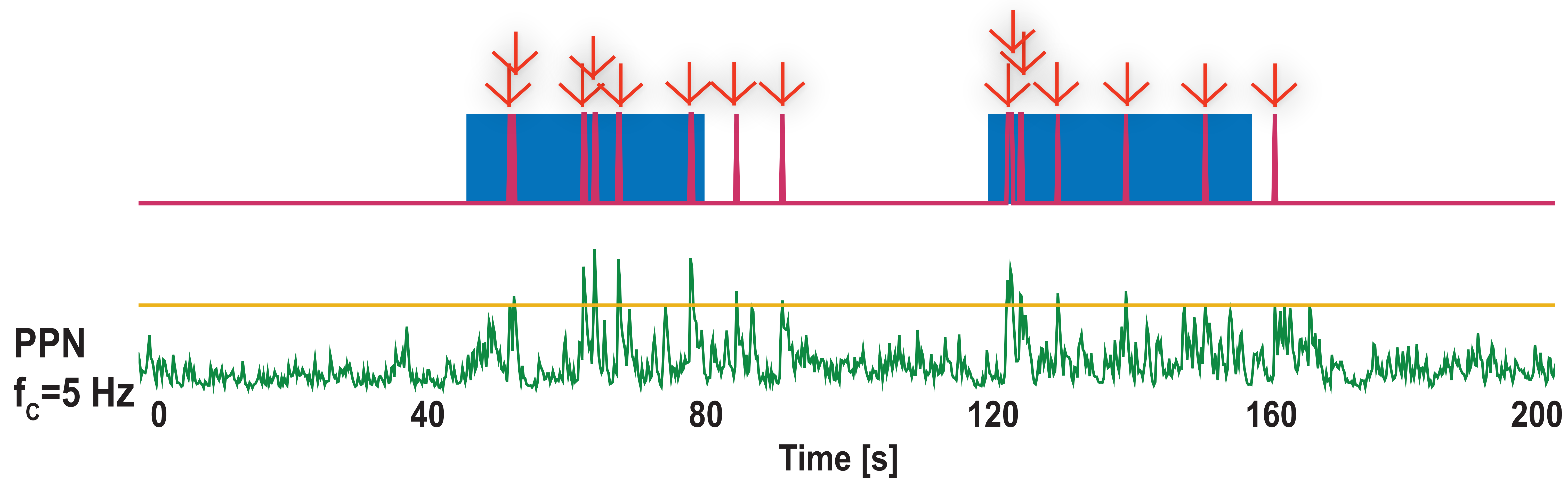**B**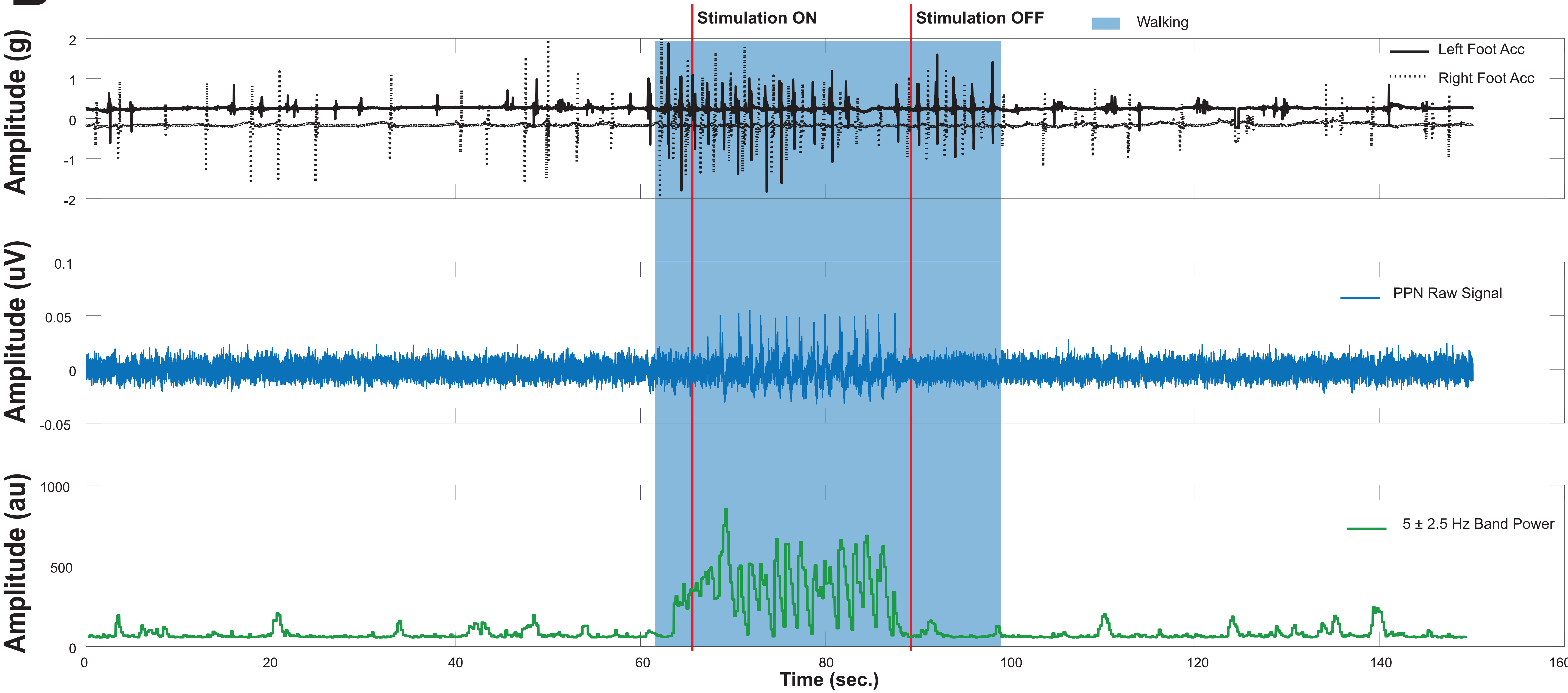

Supplement: SUPPLEMENTARY TABLE 1 — Lead locations of the active contacts. [file Data_Sheet_1.PDF]

**A**

GPi Beta Peak

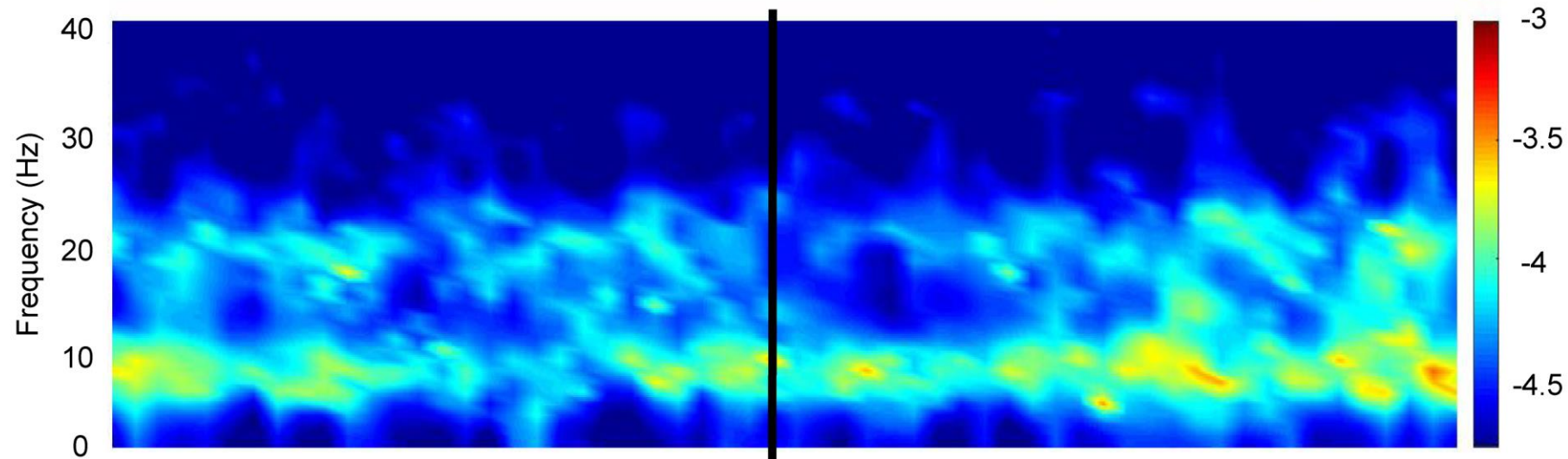**B**

PPN 1-8 Hz

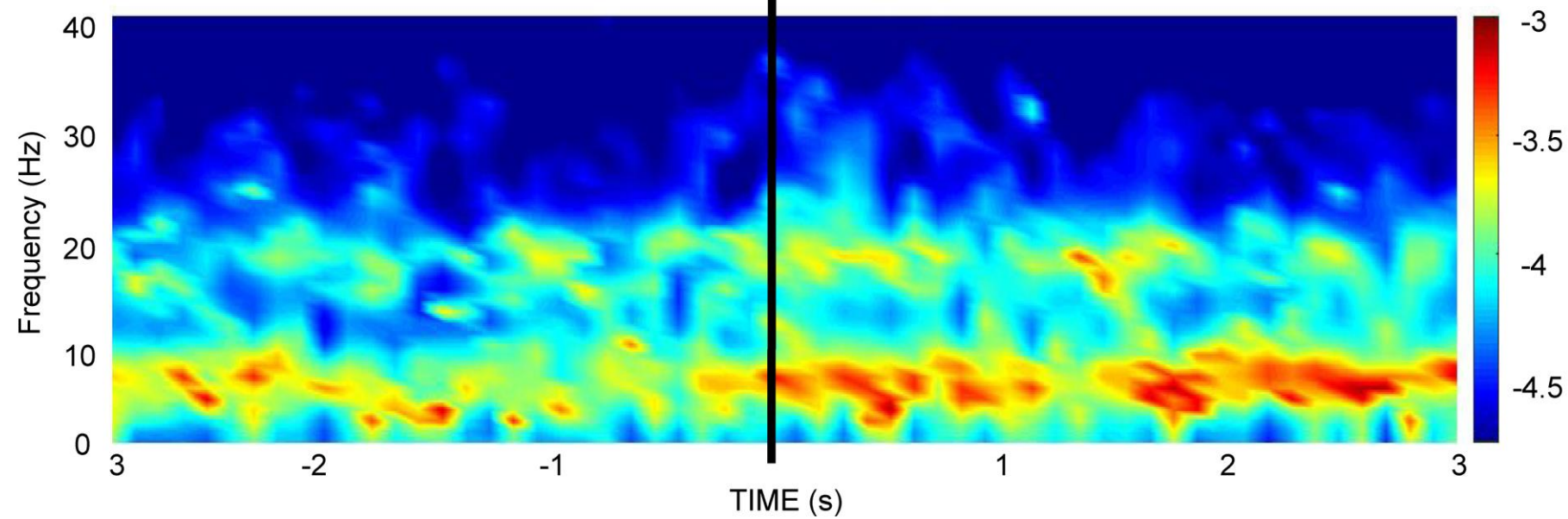

Supplement: SUPPLEMENTARY TABLE 2 — Stimulation protocol at each month. [file Data_Sheet_2.PDF]
